# Effects of adenovirus-mediated knockdown of IRAK4 on synovitis in the osteoarthritis rabbit model

**DOI:** 10.1186/s13075-021-02684-8

**Published:** 2021-12-04

**Authors:** Muzhe Li, Huiyun Li, Xun Ran, Han Yin, Xuling Luo, Zhiwei Chen

**Affiliations:** 1 Department of Orthopedic, The First Affiliated Hospital, Hengyang Medical School, University of South China, No. 69, Chuanshan Road, Hengyang City, 421001 Hunan Province China; 2 Institute of Orthopedics, The First Medical Center, Chinese PLA General Hospital, Beijing Key Lab of Regenerative Medicine in Orthopedics, Key Laboratory of Musculoskeletal Trauma and War Injuries PLA, No. 28 Fuxing Road, Haidian District, Beijing, 100853 China

**Keywords:** Interleukin-1 receptor-associated kinase 4, Synovitis, Osteoarthritis, Toll-like receptor/IL-1 receptor, Inflammation

## Abstract

**Background:**

The use of interleukin-1 receptor-associated kinase 4 (IRAK4) inhibitor as a treatment for the inflammatory joint disease is a promising method. However, its underlying mechanism in osteoarthritis (OA) remains unclear. The purpose of this study is to look into the effects of adenovirus-mediated knockdown of IRAK4 on synovitis in the OA rabbit model.

**Methods:**

Ad-shIRAK4 was injected two weeks after anterior cruciate ligament resection. Six weeks later, the rabbits were killed. The expression of IRAK4, TNFR-associated factor 6(TRAF6), TGF-activated kinase 1(TAK1), p-IKB kinase (p-IKK), p-nuclear factor kappa-B (p-NFκB), p38, and p-p38 in the synovial membrane was detected by western blot, qRT-PCR, and immunohistochemistry analysis. Immunohistochemistry was to detect the expression of IRAK4 proteins in articular cartilage. H&E staining was to assess the pathological changes of synovium and cartilage. The levels of interleukin (IL)-1β, tumor necrosis factor-α(TNF-α), and MMP-13 in the synovial fluid were measured by ELISA. X-ray and micro-computerized tomography (μCT) scans were used to assess knee joint conditions and microstructure of subchondral bone.

**Results:**

IRAK4 expression levels in synovial tissues of the OA model group exhibited a significant upward trend. Ad-shIRAK4 significantly reduced IRAK4 mRNA expression in synovium tissues. Notably, Ad-shIRAK4 suppressed the Toll-like receptor/interleukin-1 receptor (TLR/IL-1R) signaling. In addition, in the Ad-shIRAK4 treatment group, we can see less inflammatory cell infiltration and reduced hyperplasia and angiogenesis. The levels of IL-1β, TNF-α, and MMP-13 in the synovial fluid in the OA model group were significantly higher than that in the control group, which were reduced by Ad-shIRAK4 treatment. Finally, Results of HE stains, immunohistochemistry, and μCT showed that Ad-shIRAK4 treatment has a protective effect on cartilage damage.

**Conclusions:**

IRAK4 is significantly upregulated in the synovium from the osteoarthritis rabbit model. In addition, Ad-shIRAK4 reduced the expression of IRAK4 and suppressed TLR/IL-1R signaling in the synovium from the osteoarthritis rabbit model. Ad-shIRAK4 could alleviate synovitis and cartilage degradation in the osteoarthritis rabbit model, and thus alleviate the symptoms of OA and prevent the progression of OA.

**Graphical abstract:**

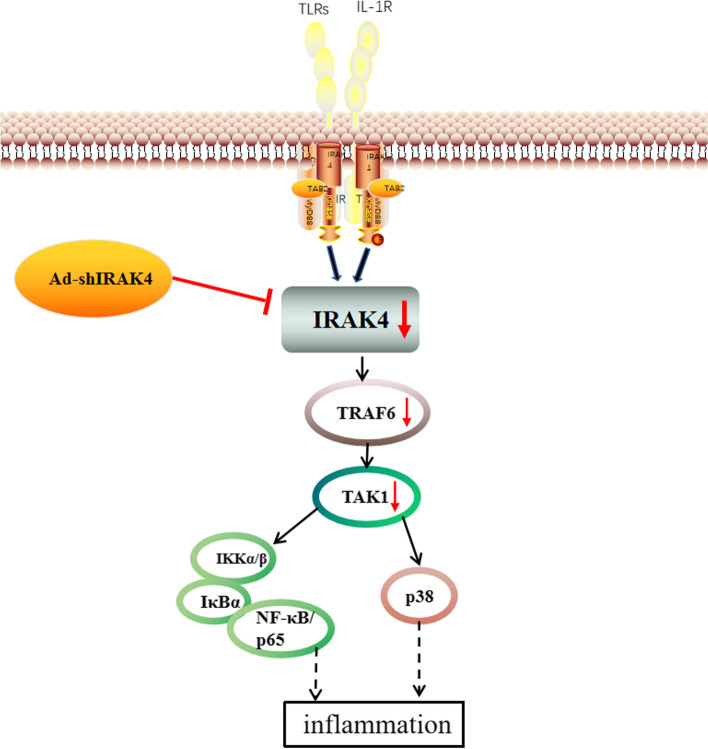

## Introduction

OA is a bone and joint degenerative condition. Synovitis, cartilage breakdown, osteophyte production, and subchondral osteosclerosis are the most common pathological symptoms. In China, the prevalence of osteoarthritis of the knee in adults over the age of 65 is as high as 85% [1, 2]. The importance of synovitis in OA has been widely recognized because it is the ultra-early manifestation of osteoarthritis and occurs throughout the course of OA [3]. Studies have pointed out that synovitis is directly related to clinical symptoms such as joint swelling and inflammatory pain [4]. Osteoarthritis synovium produces inflammatory mediators, such as IL-1β and TNF-α, which disrupt the balance between cartilage matrix degradation and repair, leading to cartilage breakdown. The destruction of cartilage, in turn, exacerbates synovitis, and the two form a vicious circle [4]. Therefore, taking synovial membrane as the target to intervene the expression of inflammatory factors is helpful to alleviate the symptoms of OA, and it will also be an important strategy to improve the pathological process of OA [5].

However, Separate intraarticular injections of biologics, such as IL-1 and TNF-αinhibitors, have been shown in recent studies to have a negative effect on OA. However, their research discovered that long-term use of these biologics may protect joints [6]. Gene therapy is the introduction of foreign genes into target cells, such as synovial cells and chondrocytes, in order to rectify or compensate for disorders caused by faulty genes and achieve therapeutic goals. Gene therapy can improve medication distribution in joints and increase drug half-life, which may be connected to the improved anti-inflammatory outcome of OA [7].

Single cytokine or growth factor alone has an insufficient effect on OA and can’t prevent the progression of OA completely, so seeking the key junctions in the main inflammatory signaling pathways to antagonize multiple inflammatory factors will be the key to the effective prevention and treatment of OA [6]. Toll-like receptor (TLR) is an important regulator of the innate immune system. A number of studies have shown that TLRs are widely expressed in synovial tissue and articular cartilage of patients with arthritis [8, 9]. TLRs can activate the immune system and release inflammatory factors, thus inducing the catabolic response of cartilage and participating in the pathological changes of OA. In addition, TLR signaling plays a key role in initiating and maintaining OA pain [10]. Interleukin-1 receptor-associated kinase 4(IRAK4) is the family member indispensable for Toll-like receptor/IL-1 receptor (TLR/IL-1R) signaling, playing an important role in the initiation and regulation of inflammatory diseases [11]. Recent studies have shown that mutations in the IRAK-4 gene are associated with various autoimmune diseases [10]. In addition, tumor cells promote the immune escape of tumor cells by inhibiting IRAK4 signaling [11]. However, the role of IRAK4 in synovitis and its connection with the pathogenesis of OA is not completely clear. In this study, we compared the expression of IRAK4 in normal synovium with that in OA synovium. Furthermore, we investigated if adenovirus-encoded short hairpin (sh) RNA (Ad-shIRAK4) reduced the expression of IRAK4 and suppressed TLR/IL-1R signaling in synovium from the osteoarthritis rabbit model. Finally, we determine if Ad-shIRAK4 alleviated the degree of synovitis, further limiting OA development.

## Methods

### Experimental design

A total of 31 New Zealand white rabbits (2.0–2.5 kg) were obtained from the Laboratory Animal Center. The study was approved by the Medical Ethical Committee. Six rabbits were used to test the effectiveness of the osteoarthritis rabbit model by dissection of the anterior cruciate ligaments in the right knees. Twenty-five rabbits were randomly divided into five groups of five rabbits each. They were the sham operation group, OA model group, adenovirus empty carrier group (1 × 10^8^ pfu, Honor Gene), adenovirus low titer group (1 × 10^8^ pfu, Honor Gene), adenovirus high titer group (1 × 10^9^ pfu, Honor Gene).

Rabbits were anesthetized with pentobarbital (30 mg/kg). After the rabbit's consciousness disappeared, the right knee joint was cut through a median knee incision. The patella is turned laterally to expose the internal structure of the knee joint, then the anterior chiasma primary band is sought and dissected. The cartilage surface of the knee joint should not be damaged during the operation. The knee joint was rinsed with normal saline 3 times and the wound was sutured layer by layer. In the sham group, only the joint space was cut open. After surgery, the rabbits were kept in fixed, individual cages and closely monitored for infection and other complications. Sticks were used to motivate all the rabbits to exercise for 2 hours a day. Two weeks later, the sham and OA model groups received 300 μl of 0.9% physiological saline by intra-articular injection. Adenovirus empty carrier group received 1 × 10^8^ pfu adenovirus empty carrier (Honor Gene). Adenovirus low titer group received 1 × 10^8^ pfu adenovirus-mediated shIRAK4 (ad-shIRAK4, Honor Gene). Adenovirus high titer group received 1 × 10^9^ pfu ad-shIRAK4 (Honor Gene). The intra-articular injection was repeated 10 days later. Six weeks after adenovirus injections, rabbits were killed by air embolization, and articular fluid, synovium, and knee joints were stored at – 80 °C until analysis.

### Morphologic observations

The tibial plateau and the femoral condyles were observed and photographed. The scoring system for macroscopic grading of cartilage damage was segmented into normal (0), surface roughening (1), fibrillation and fissures (2), small erosions down to the subchondral bone (3), and large erosions down to the subchondral bone (4) [12].

### Image assessment

The knee joint images of rabbits were scanned by micro-CT equipment and reconstructed (GE Medical, Milwaukee, WI, USA). The scanned images from each group were evaluated at the same thresholds to allow 3-dimensional structural rendering of each sample. The evaluation indicators include bone volume/tissue volume ratio (BV/TV), trabecular number (Tb. N), and the growth rate of subchondral sclerosis [13]. The growth rate of subchondral sclerosis was calculated by the volume ratio of the OA model group alone or, adenovirus carrier group to the control group [12, 14]. The knee joint condition was evaluated by X-ray images from both anterior and lateral views and further graded blindly according to the Kellgren-Lawrence (K-L) grading system.

### Enzyme-linked immunosorbent assay (ELISA)

The levels of interleukin-1β(IL-1β), tumor necrosis factor-α(TNF-α), and matrix metalloproteinase-13(MMP-13) in the articular fluid were evaluated using the rabbit IL-1β ELISA kit (Jianglai, E72059), rabbit TNF-α ELISA kit (Jianglai, E72292), and rabbit MMP-13 ELISA kit (Jianglai, E72146). The measurement was carried out in accordance with the product manual.

### RNA isolation and quantitative real-time PCR (qRT-PCR)

Total RNA was extracted from synovial tissue of the knee joint using TRIzol (Ambion, USA). The concentration and purity of the extracted RNA were measured by spectrophotometer. Then, the RNA was reverse-transcribed, and the resulting cDNA was amplified by PCR. PCR reactions (per well: 1 μL CDNA, 0.5 μL each of forward and reverse primers, 10 μL 2×RealStar Green Fast Mixture (with ROX), 8 μL ddH2O) was performed using an SYBR Green real-time PCR kit (GenStar) and Archimed-X6 qRT-PCR system. All experiments were repeated at least thrice, and the amplification signals from individual target genes were normalized to that of glyceraldehyde-3-phosphate dehydrogenase (GAPDH). The primer sequences were shown in Table 1.

**Table 1:** Primers

Gene	Direction of primer	The primer sequences(5′-3′)
**IRAK4**	FORWARD	CTGTGGATGAACACCGTGAACCTC
	REVERSE	GGGCTCAGCATCACTCATCTTCG
**TRAF6**	FORWARD	CCTGGATTCTACACGGGCAAACC
	REVERSE	AGGAAGGTGGCTGTCATACTCTCC
**IKK**	FORWARD	GCAGACCGTGAACATCCTCT
	REVERSE	TCCAGGACAGTGAACGAGTG
**NFκB**	FORWARD	CACTGCCGAGCTCAAGATCTGCC
	REVERSE	GTCGGCGTACGGAGGAGTCCG
**IL-1β**	FORWARD	TACAACAAGAGCTTCCGGCA
	REVERSE	GGCCACAGGTATCTTGTCGT
**GAPDH**	FORWARD	TTGTCGCCATCAATGATCCAT
	REVERSE	GATGACCAGCTTCCCGTTCTC

### Traditional western blot

The synovial tissue samples were removed from the − 80 °C refrigerator. After the tissues were ground with liquid nitrogen, these samples were lysed for 1.5 h with RIPA lysate containing 0.1% PMSF in the icebox. Then, the cracked samples were centrifuged in a centrifuge at 12,000 RPM at 4 °C for 20 min. The supernatant is the total protein solution. The bicinchoninic acid method was used to detect the protein concentration and calculate the loading volume. Then, separating gel and concentrated gel were prepared and electrophoresis began after loading samples in a certain order. After electrophoresis to the gel substrate, the gel was removed and transferred to the PVDF membrane. The membrane and the first antibody (1:1000) were incubated overnight at 4 °C. On the second day, the membrane was incubated in the second antibody (1:300) at room temperature for 1 h. Finally, the gray value is detected after the PVDF membrane was exposed to 200 μL ECL solution.

### Automated western blot

The total protein was extracted from synovial tissue by referring to the above method and the bicinchoninic acid method was used to detect the protein concentration. The simple western immunoblots were conducted on a JESS/Wes (ProteinSimple) using the Size Separation Master Kit with Split Buffer (12–230 KDa) according to the manufacturer’s standard instruction. The following antibodies are used: IRAK4 (CST); TNFR-associated factor 6 (TRAF6, Bioss); TGF beta-Activated Kinase 1 (TAK1, Bioss); IKB kinase (p-IKK, Bioss); nuclear factor kappa-B (p-NFκB p65, Bioss); β-actin (Abcam); p38 (Bioss); and p-p38 (Bioss).

### Histology analysis and immunohistochemistry

After the animals were killed, the patellar ligament was severed. The joint cavity was opened, and the synovial tissues on both sides of the joint capsule was quickly removed with tissue forceps. Synovial tissues of the rabbits were fixed with paraformaldehyde overnight (4%), paraffin embedding, and sagittal sections (cut at approximately 7 μm). To assess the degree of synovitis, sections of the sample were stained with Hematoxylin and Eosin (H&E) (Solarbio, China) by following the manufacturer’s standard instruction. The synovitis pathology score was determined primarily by three factors: the degree of proliferation of the synovitis lining layer, the degree of inflammation of the lining layer, and angiogenesis [15]. Synovitis severity was scored [(severity score = sum of pannus formation score (0–3) + synovial lining hyperplasia score (0–3) + sub-synovial inflammation score (0–3)].

Immunohistochemistry analysis was performed by using antibodies against IRAK4, TRAF6 and TAK1to observe their deposition in the synovial membrane. Immunohistochemistry was to detect the expression of IRAK4 proteins in articular cartilage. In brief, sections were incubated overnight at 4 °C with the first antibodies. Then, sections were washed thrice with PBS and incubated in the second antibody for 1h. Finally, sections were exposed to DAB solution and were captured after the section is sealed with neutral resins. Dark brown is considered a positive expression. The positive area was quantified by ImageJ software.

To assess the articular surface, osteochondral samples from the femur and tibia were fixed in 4% paraformaldehyde for 48 h and then decalcified for 7 weeks using ethylenediaminetetraacetic acid (EDTA) solution. Following paraffin embedding, the samples were sliced into 7-m slices and stained with H&E. Cartilage tissue was scored according to Mankin scoring criteria, and the score of each specimen was the mean value of cartilage sections [16].

### Statistical analysis

All data were expressed as means ±standard deviation (SD) for a minimum of *n* = 3. Statistical analysis was performed using the SPSS 22.0 statistical software. Statistical significance was indicated by a *p* value < 0.05. Comparisons between the two groups were conducted t-test; Comparisons between the three groups were conducted one-way ANOVA.

## Results

### IRAK4 is upregulated in the synovium from the osteoarthritis rabbit model

**Fig. 1:**
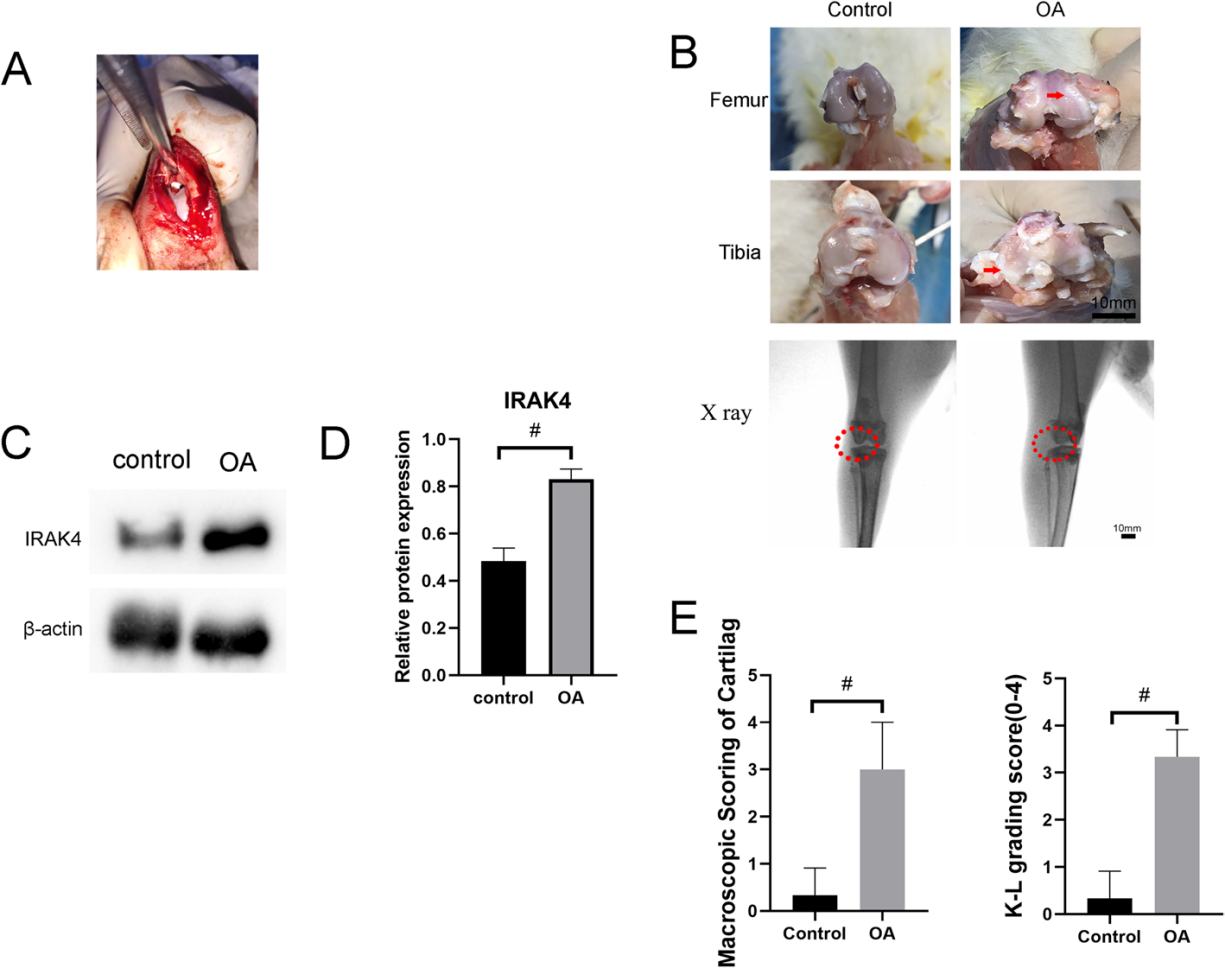
IRAK4 is upregulated in the synovium from the osteoarthritis rabbit model. **A** Anterior cruciate ligament resection was performed in New Zealand white rabbits (2.0–2.5 kg). **B C**artilage damage was observed in rabbits 6 weeks after anterior cruciate ligament resection. Images of X-rays were obtained in rabbits 6 weeks after anterior cruciate ligament resection. **C** The protein expression levels of IRAK4 were detected by western blot. **D** Quantification of the relative protein expression via ImageJ software, β-actin was served as the internal control. **E** Macroscopic scoring of cartilage and K-L grading for X-ray. Data are expressed as the mean ± SD. Statistical analysis was performed by T tests, #*P* <0.05, compared between control and OA model groups. *n*=3 per group

To verify the effectiveness of the osteoarthritis model, we performed anterior cruciate ligament resection in New Zealand white rabbits (2.0–2.5 kg) (Fig. 1A). We carried out a macroscopic observation to assess knee joint damage including articular cartilage degradation, synovial tissue hyperplasia 6 weeks after surgery. As expected, the semi-quantification score of cartilage damage for femoral and tibial condyles (Fig. 1B) showed that compared with the sham operation group, the OA model group showed that joint space was severely narrow. In addition, the surface of the knee joint was not smooth, with obvious defects and osteophytes, presenting evident signs of osteoarthritis. (Fig. 1E) Knee joint conditions (red circle in Fig. 1B) were evaluated by X-ray imaging. Kellgren-Lawrence grading revealed that the control group was at Grade 0 and the OA group was at Grade 3–4 (Fig. 1E) To compare the expression of IRAK4 in normal synovium with that in OA synovium, we found that compared with the sham operation group, IRAK4 expression levels in synovial tissues of the OA model group exhibited a significant upward trend through quantitative analysis of protein. (Fig. 1C, D)

### Ad-shIRAK4 alleviated the degree of synovitis in the osteoarthritis rabbit model

**Fig. 2:**
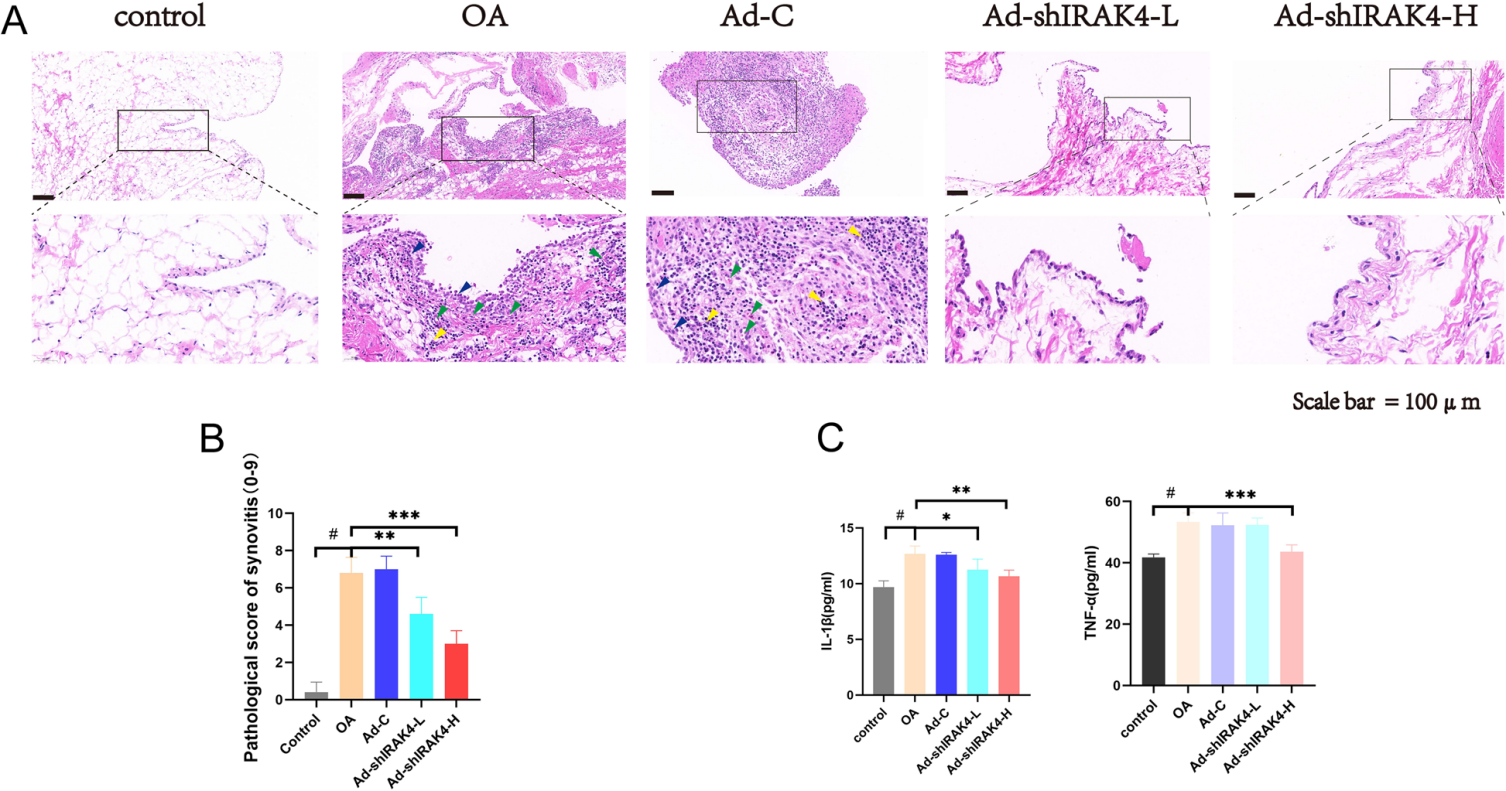
Ad-shIRAK4 alleviated the degree of synovitis in the osteoarthritis rabbit model. **A**, **B** H&E staining was applied to assess histopathological changes in the synovial membrane. Blue arrowheads indicate the enlarged lining cell layer, green arrowheads indicate angiogenesis and yellow arrowheads indicate inflammatory cell infiltration. The pathological score of synovitis was used to quantitatively assess the severity of synovitis. **C** The levels of IL-1β and TNF-α in the synovial fluid were measured by ELISA. Data are expressed as the mean ± SD. Statistical analysis was performed by one-way ANOVA, ^#^*P* < 0.05 vs. control group; *P <0.05, **P <0.01, ***P <0.0001 vs. OA model group. *n*=5 per group

Next, we performed a series of tests to determine if Ad-shIRAK4 lowers the degree of synovitis in the osteoarthritis rabbit model. Our macroscopic assessment of the synovium found that the synovial membrane is thickened and dark red with obvious hyperemia on the synovial surface in the OA model group, which were alleviated by Ad-shIRAK4 treatment, although still more serious than that in the control group. The pathological score of synovitis was mainly evaluated from three aspects: the degree of proliferation of the synovitis lining layer, the degree of inflammation of the lining layer, and angiogenesis [15]. As shown in the result of H&E, compared with the control group, synovial hyperplasia severity, inflammatory cell infiltration, and angiogenesis were significantly aggravated in the OA model group. However, in the Ad-shIRAK4 treatment group, we can see less inflammatory cell infiltration and reduced hyperplasia and angiogenesis, although still higher than that in basal levels (Fig. 2A). The quantified results of the pathological score of synovitis are shown in Fig. 2B. IL-1β and TNF-α, two important upstream inflammatory cytokines, are the main components of the synovial fluid of OA and are positively correlated with the pathological manifestations of OA [17, 18]. Results show that the levels of IL-1β and TNF-α in the synovial fluid in the OA model group were significantly higher than that in the control group, which were reduced by Ad-shIRAK4 treatment (Fig. 2C). These data suggest that Ad-shIRAK4 could alleviate the degree of synovitis in the osteoarthritis rabbit model.

### Ad-shIRAK4 reduced the expression of IRAK4 and suppressed TLR/IL-1R signaling in the synovium from the osteoarthritis rabbit model

**Fig. 3:**
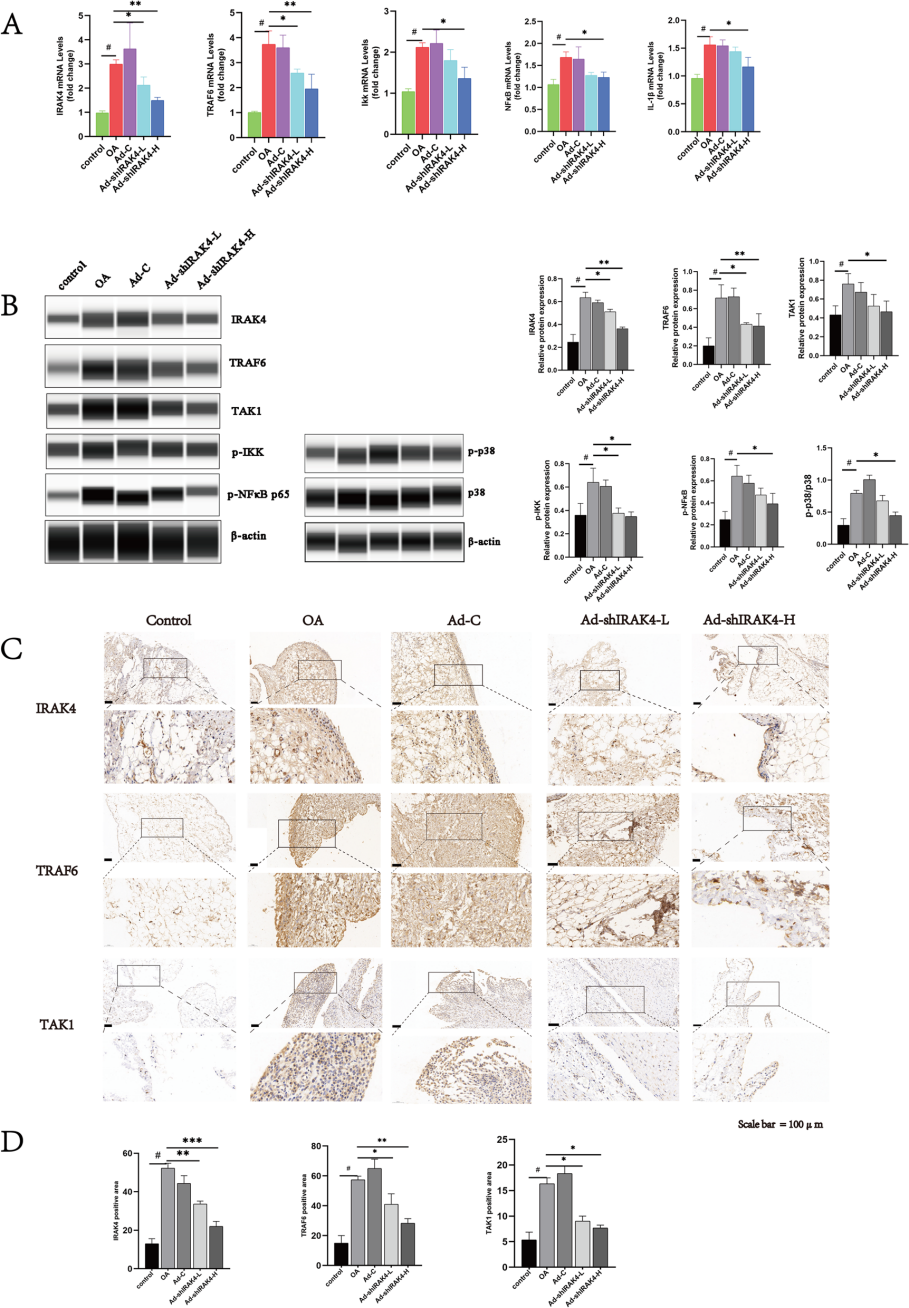
Ad-shIRAK4 reduced the expression of IRAK4 and suppressed TLR/IL-1R signaling in the synovium from the osteoarthritis rabbit model. **A** IL-1β, IRAK4, TRAF6, IKK, and NFκB mRNA expression in synovial tissue was detected by qRT-PCR. **B** IRAK4, TRAF6, TAK1, p-IKK, p-NFκB, p38, and p-p38 protein expression in synovial tissue was detected by an automated western blot. Quantification of the relative protein expression via Image-J software, β-actin was served as the internal control. **C** Immunohistochemistry to identify IRAK4, TRAF6, and TAK1 in synovial tissue. **D** Positive staining for IRAK4, TRAF6, and TAK1were quantified by ImageJ software. Data are expressed as the mean ± SD. Statistical analysis was performed by one-way ANOVA, ^#^*P* < 0.05 vs. control group; *P <0.05, **P <0.01, ***P <0.0001 vs. OA model group. *n*=3 per group. OA, OA model group; Ad-C, adenovirus empty vector group; Ad-shIRAK4-L, adenovirus low titer group; Ad-shIRAK4-H, adenovirus high titer group

To investigate the in vivo functions of IRAK4, we knock it down in synovium tissues via intra-articular injection of Ad-shIRAK4. Results of PCR showed that Ad-shIRAK4 significantly reduced IRAK4 mRNA expression in synovium tissues at 8 weeks after anterior cruciate ligament resection (Fig. 3A). Notably, Ad-shIRAK4 suppressed the TLR/IL-1R signaling, so it also reduced mRNA expression of interleukin (IL)-1β, TNFR-associated factor 6(TRAF6), IKB kinase (IKK), and nuclear factor kappa-B (NFκB) (Fig. 3A). Furthermore, by western blot, we found that, compared with the control group, IRAK4 protein expression significantly increased in synovium from the osteoarthritis rabbit model. However, Ad-shIRAK4 significantly decreased IRAK4 protein expression (Fig. 3B). Similarly, Ad-shIRAK4 significantly decreased protein expression of TRAF6, TAK1, p-IKK, p-NFκB, p38, and p-p38 in synovium tissues at 8 weeks after anterior cruciate ligament resection (Fig. 3B). Using IHC analysis, we also found that IRAK4 is upregulated in synovium from the osteoarthritis rabbit model. Ad-shIRAK4 reduced the expression of IRAK4 and suppressed TLR/IL-1R signaling in the synovium from the osteoarthritis rabbit model (Fig. 3C, D)

### Ad-shIRAK4 limited OA development and delayed OA progression in the osteoarthritis rabbit model

**Fig. 4:**
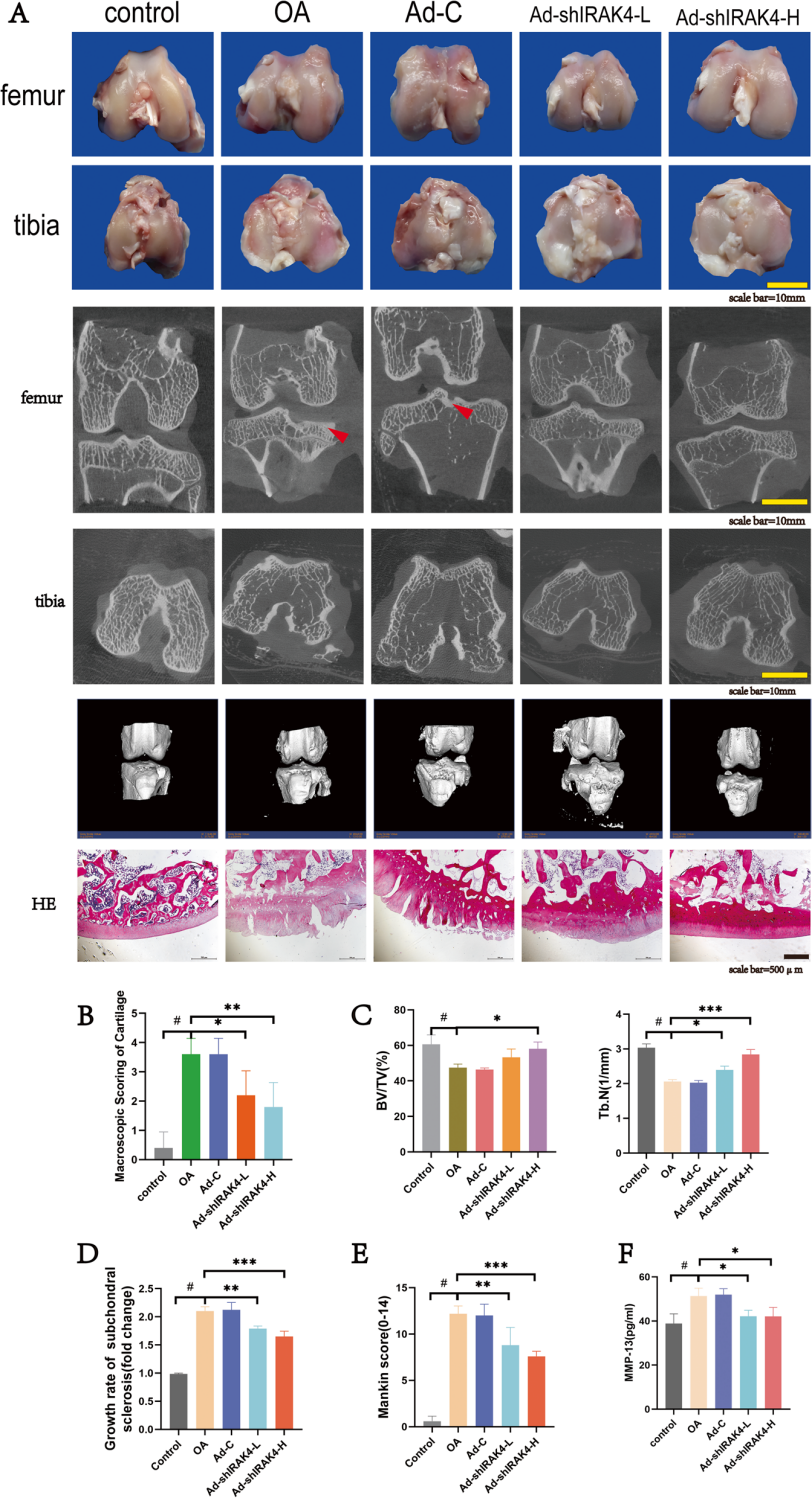
Ad-shIRAK4 limited OA development and delayed OA progression in the osteoarthritis rabbit model. **A**, **B** Cartilage damage was observed in rabbits 8 weeks after anterior cruciate ligament resection. the treatment with Ad-shIRAK4 significantly alleviated the cartilage damage. **A**, **C**, **D** Two-dimensional and three-dimensional images of micro-computerized tomography (μCT) scans were obtained 8 weeks after anterior cruciate ligament resection. **A**, **E** H&E stains were used to assess cartilage pathology. **F** ELISA was used to detect the level of MMP-13 in synovial fluid. Data are expressed as the mean ± SD. Statistical analysis was performed by one-way ANOVA, ^#^*P* < 0.05 vs. control group; *P < 0.05, **P <0.01 vs. OA model group. *n*=5 per group

**Fig. 5:**
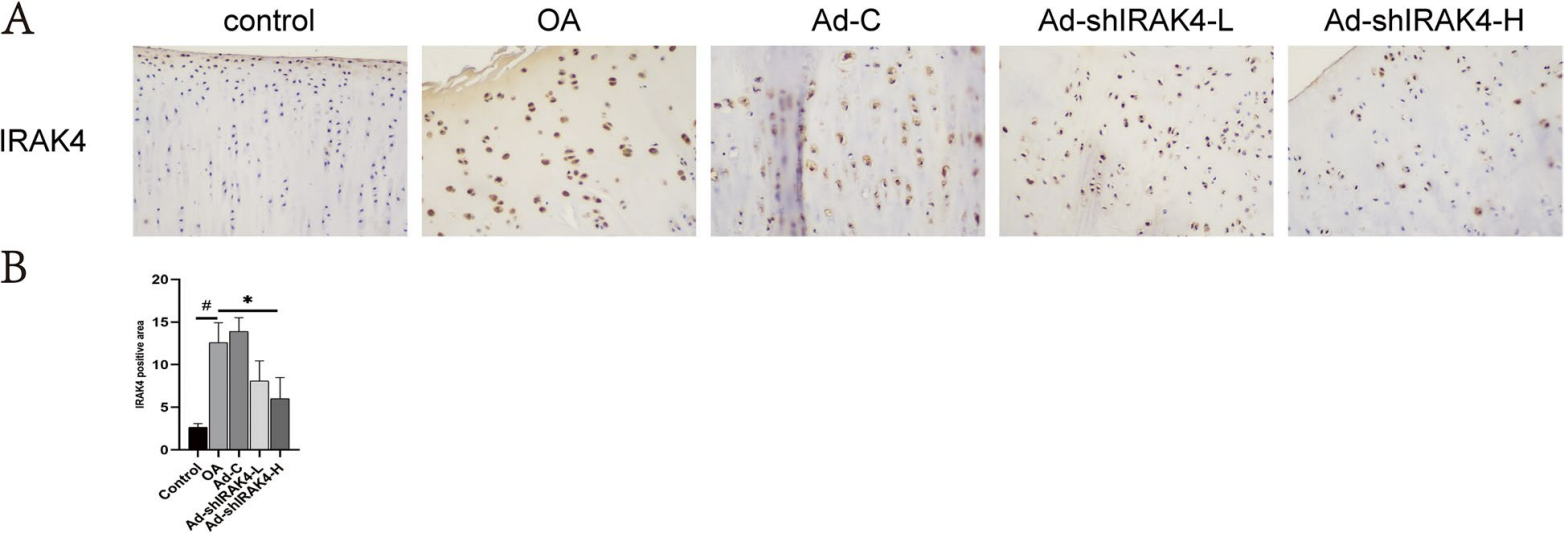
Ad-shIRAK4 attenuates the expression of inflammatory signaling in articular cartilage from the osteoarthritis rabbit model. **A** Immunohistochemistry analysis was used to assess IRAK4 in cartilage. Data are expressed as the mean ± SD. Statistical analysis was performed by one-way ANOVA, ^#^*P* < 0.05 vs. control group; *P < 0.05 vs. OA model group. *n*=3 per group

To further investigate the effect of Ad-shIRAK4 on OA development and progression, we carried out a series of experiments. Firstly, OA-like lesions were observed in rabbits 8 weeks after anterior cruciate ligament resection at the macroscopic level (Fig. 4A). The macroscopic score of cartilage for femoral and tibial condyles showed that the treatment with Ad-shIRAK4 significantly alleviated the cartilage damage (Fig. 4B). Next, Results of the tibial plateau with μCT showed that, compared with the sham operation group, subchondral bone mass was significantly increased in the OA model group. In contrast, subchondral bone mass was significantly reduced by treatment with Ad-shIRAK4 in the rabbits 8 weeks after anterior cruciate ligament resection (Fig. 4A, D) In addition, μCT analysis of femoral condyle showed that compared with OA group, BV/TV and Tb. N were significantly increased in the Ad-shIRAK4 group (Fig. 4A, C). Results of H&E staining of cartilage also showed that the treatment with Ad-shIRAK4 can improve cartilage structure, reduce chondrocyte apoptosis, and delay the degradation of the extracellular matrix (Fig. 4A, E). MMP-13 is a major enzyme targeting cartilage degradation. Compared with other MMPs, MMP13 expression is more restricted to connective tissue. It degrades not only type II collagen in cartilage, but also proteoglycan, type IV and type IX collagen, osteonectin, and basement membrane proteoglycan in cartilage [19]. The level of MMP-13 in synovial fluid was analyzed by ELISA. we founded that the levels of MMP-13 in the synovial fluid in the OA model group were significantly higher than that in the control group, which were reduced by ad-shIRAK4 treatment (Fig. 4A, F). Finally, immunohistochemistry analysis was used to detect the expression of inflammatory signaling in articular cartilage. Immunohistochemical experiments showed that IRAK4 proteins were increased in the OA model group. The IRAK4 proteins were remarkably decreased in articular cartilage after injection of ad-shIRAK4 (Fig. 5). These data suggest that ad-shIRAK4 limited OA development and delayed OA progression in the osteoarthritis rabbit model.

## Discussion

OA, a chronic and progressive synovial joint disease, remains common and difficult to cure. In recent years, the role of inflammaory factors in the occurrence and development of OA has attracted widespread attention. OA is no longer considered a degenerative disease, but an inflammatory disease with the participation of inflammatory genes [20]. Prior to the onset of early cartilage degeneration, the synovial membrane shows significant changes, including synovial lining thickening, macrophage infiltration, stromal angiogenesis, and inflammatory cytokine production [21]. Histological and radiographic studies confirm the high incidence of synovitis at all stages of osteoarthritis. In addition, several studies have shown that synovitis and its pro-inflammatory factors are associated with knee pain, swelling, and dysfunction, and may even be an independent factor in the onset and structural progression of osteoarthritis [4, 22, 23]. As a result, using the synovial membrane as a target to interfere in the production of inflammatory factors is beneficial in alleviating OA symptoms and will be an essential strategy in improving the pathological process of OA [5].

Researches have shown that a single cytokine or growth factor alone has an insufficient effect on OA and cannot prevent the progression of OA completely, so seeking the key junctions in the main inflammatory signaling pathways to antagonize multiple inflammatory factors will be the key to the effective prevention and treatment of OA [6]. IRAK4 is essential for TLR/IL-1R signaling, playing a critical role in the initiation and control of TLR/IL-1R-mediated inflammatory disorders [11].

IRAK4 inhibitors have been proven in numerous trials to be a viable therapeutic option for inflammatory joint cells by cutting crucial linkages between them. The ability of IRAK4 inhibitor therapy to restrain the migration of joint F480+iNOS+ Ms, vimentin+ fibroblasts, and CD3+ T cells, in addition to negating the expression of a wide range of monokines, including IL-12, MIP2, and IRF5, and Th1/Th17 lymphokines, explains the superior efficacy of IRAK4 inhibitor therapy over anti-TNF or anti-IL-6R therapy in miR-Let7 [24]. Loss of IRAK4 kinase activity inhibited TLR ligand and IL-1-induced cytokine and NF-κB signaling [25]. Rheumatoid arthritis (RA) is a common inflammatory illness characterized by persistent erosive arthritis that results in gradual bone loss, eventually leading to joint deformity and dysfunction. To explore how IRAK4-Interferon Regulatory Factor (IRF) signals regulate the expression of inflammatory mediators and influence neuropathology, Ngwa C et al. found that inhibition of IRAK4 phosphorylation inhibited the inflammatory response of microglia cells and increased the length of neurite processes and neuronal activity after ischemia [26]. Similarly, knocking down IRAK4 in microglia decreased the IRAK4 expression and inhibited the activation of NF-ĸB and the downstream production of proinflammatory factors, thus reducing the intracerebral hemorrhage (ICH) induced microglia activation and inflammatory cytokines release [27]. In addition, IRAK4 promotes inflammatory osteolysis by activating osteoclasts and inhibiting the formation of foreign body giant cells (FBGC). IRAK4 deficiency restores the formation of FBGC and promotes the expression of M2 macrophage markers, thereby inhibiting inflammation [28]. The role of IRAK4 in synovitis and its connection with the pathogenesis of OA is not clear. Therefore, it is worth further discussion.

This experiment explored the key role of IRAK4 in the osteoarthritis rabbit model. a classic OA model was made by anterior cruciate ligament amputation. This model can not only avoid the unknown mechanism of action of the modeled drugs in joint injection but also avoid the influence of the modeled drugs on the therapeutic drugs. We found that the model was effective at inducing synovitis and cartilage damage after 6 weeks. HE staining results of synovitis and cartilage showed that Ad-shIRAK4 alleviated the degree of synovitis in the osteoarthritis rabbit model, and had a protective effect on cartilage damage. μCT analysis of femoral condyle also showed that BV/TV and Tb. N was significantly increased in the Ad-shIRAK4 group. This means that Ad-shIRAK4 can also play a role in protecting OA subchondral bone microstructure damage.

TLRs are an important class of pattern recognition receptors (PRRs) in the innate immune system. Through the analysis of pathogen-associated molecular patterns (PAMPs) and endogenous danger signal-associated molecular patterns (DAMPs), the NF-κB signal transduction pathway was activated, which caused the secretion of cytokines and inflammatory mediators, and initiated the innate immune response, thereby inducing or aggravating OA [29, 30]. TLR intracellular domain and IL-1R intracellular structure form a homologous double chain known as the TLR/IL-1R superfamily [31]. TLR/IL-1R binds to the ligand and phosphorylates IRAK-4, activating it. TRAF6 is recruited by phosphorylated IRAK4. TRAF6 was dissociated after ubiquitination and recruited TAK1 and TAB. The activated TAK1 complex activates IKK through phosphorylation. Activated IKK phosphorylates the inhibitor protein of NF-κB (IκBα), which releases NF-κB and translocates into the nucleus, inducing increased expression of a series of inflammatory factors [32]. As an another key downstream signaling pathways of IRAK4, p38 signaling pathways involved in the synthesis of articular cartilage matrix metalloproteinases, the production of inflammatory cytokines, maintain and differentiation in the phenotype of chondrocytes, hypertrophy and calcification of chondrocytes, closely related to the apoptosis of chondrocytes, plays a crucial role in the process of articular cartilage degeneration [33]. The results of this study show that Ad-shIRAK4 reduced the expression of IRAK4 and suppressed TLR/IL-1R signaling in the synovium from the osteoarthritis rabbit model.

In our study, local intraarticular injection of adenovirus vectors was used. Synovial tissue is relatively loose, and synovial cells on the synovial surface are more easily transfected than chondrocytes wrapped in a dense cartilage matrix. Synovial cells are therefore a major target for adenovirus transfection. However, evidence has shown that, IL-1R accessory proteins like MyD88, IRAK1, TRAF6, and NF-κB have been detected in OA chondrocytes [34]. Our data show that there is cartilage matrix destruction in the OA knee, resulting in exposure of chondrocytes. Our experimental results also showed that ad-shIRAK4 could significantly reduce the expression of inflammatory factors in chondrocytes. Therefore, we believe that ad-shIRAK4 also has a direct protective effect on chondrocytes.

Synoviocytes are found in the synovium lining layer, which is made up of several types of macrophage-like synoviocytes (MLS) and fibroblast-like synoviocytes (FLS). FLS, as the major cellular component of the synovial membrane, is not only significant in synovial inflammation but is also linked to cartilage destruction and discomfort in arthritis sufferers [21]. The NF-B protein family was found in significant concentrations in OA-FLSs and joint fluid [35]. Nair A et al investigated whether a TLR-2 or TLR-4-stimulating factor in synovial fluid (SF) from OA patients might cause inflammatory activation of FLS and discovered that SF sensitises FLS to react to inflammatory stimuli [36]. In addition, research has confirmed that TLR2, TLR4, and MyD88 mRNA expression was increased in FLS from patients with OA [37]. In OA-FLS, MAPK signaling pathways are also substantially active, resulting in the release of a large number of inflammatory factors [38]. The evidence presented above implies that FLS is the primary target cell. When compared to normal synovial tissue, the number of MLS, mostly M1-type macrophages, was considerably higher in OA patients’ synovial tissue [39, 40]. TNF-α and IL-1β are two inflammatory cytokines produced by MLS that are implicated in the development of OA. Meanwhile, these inflammatory cytokines can activate macrophages, resulting in a vicious cyclical process [41]. Through the NF-κB pathway, MLS can create a range of pro-inflammatory mediators, and the release of these mediators can result in FLS activation [42]. When macrophages in synovial cells of patients with knee arthritis were eliminated, not only were the inflammatory cytokines generated by them greatly down-regulated, but so were the cytokines mostly produced by FLS, such as IL-6, IL-8, MMP-1, and MMP-3. Through inflammatory mediators, MLS can modulate FLS and contribute in the OA disease process [40]. Numerous studies have revealed that NF-κB and MAPK signaling are active in OA-MLS and play a crucial role in the onset and progression of macrophage-mediated OA [43–45]. As a result, ad-shIRAK4 may suppress M1-type macrophages and restore the M1/M2 balance, relieving synovitis. Furthermore, IL-1β and TNF-αsuppress chondrogenesis in human mesenchymal stem cells via NF-κB-dependent pathways. As a result, by targeting MSCs, ad-shIRAK4 can also enhance cartilage repair [46]. OA synovitis is typically persistent and dominated by macrophages. As a result, the actions of ad-shIRAK4 on immune cells including neutrophils and lymphocytes may have only a limited impact in OA [47]. Adipocytes are identified in lower numbers in synovial tissue during the development and progression of arthritis [48]. The expression of TLR/IL-1R signaling in adipocytes is not known. There are also some deficiencies in this experiment. First, this study did not compare the efficacy of commonly used anti-inflammatory drugs such as cyclooxygenase-2. In addition, the variability of the MLS and FLS response to ad-shIRAK4 in OA has yet to be studied at the single-cell level. Finally, this study also lacks relevant experiments to verify whether Ad-shIRAK4 reduces OA pain sensitivity. There may be a long way to go to reach the final conclusion.

## Conclusions

IRAK4 is significantly upregulated in the synovium from the osteoarthritis rabbit model. Ad-shIRAK4 reduced the expression of IRAK4 and suppressed TLR/IL-1R signaling in the synovium from the osteoarthritis rabbit model. Ad-shIRAK4 could alleviate synovitis and cartilage degradation in the osteoarthritis rabbit model and thus alleviate the symptoms of OA and prevent the progression of OA.

## Data Availability

We confirm that all relevant data are available from the authors

## Abbreviations

OA: Osteoarthritis
ad-shIRAK4: Adenovirus-mediated shIRAK4
μCT: Micro-computerized tomography
IRAK4: Interleukin-1 receptor-associated kinase 4
TRAF6: TNFR-associated factor 6
TAK1: TGF-activated kinase 1
IKK: IKB kinase
NFκB: Nuclear factor kappa-B
TLR/IL-1R: Toll-like receptor/interleukin-1 receptor
IL-1β: Interleukin-1β
TNF-α: Tumor necrosis factor-α
MMP-13: Matrix metalloproteinase-13
BV/TV: Bone volume/tissue volume ratio
Tb. N: Trabecular number
K-L: Kellgren-Lawrence
EDTA: Ethylenediaminetetraacetic acid
SD: Standard deviation
RA: Rheumatoid arthritis
IRF: Interferon Regulatory Factor
ICH: Intracerebral hemorrhage
FBGC: Foreign body giant cells
PRRs: Pattern recognition receptors
PAMPs: Pathogen-associated molecular patterns
DAMPs: Danger signal-associated molecular patterns
TAB: TAK1 binding proteins
COX-2: Cyclooxygenase-2
MLS: Macrophage-like synoviocytes
FLS: Fibroblast-like synoviocytes
